# Decrease of GSK-3β Activity in the Anterior Cingulate Cortex of *Shank3b*^−/−^ Mice Contributes to Synaptic and Social Deficiency

**DOI:** 10.3389/fncel.2019.00447

**Published:** 2019-10-23

**Authors:** Mengmeng Wang, Xinyan Liu, Yilin Hou, Haifeng Zhang, Junjun Kang, Fei Wang, Youyi Zhao, Jing Chen, Xufeng Liu, Yazhou Wang, Shengxi Wu

**Affiliations:** ^1^Department of Neurobiology, Institute of Neurosciences, School of Basic Medicine, Fourth Military Medical University, Xi’an, China; ^2^Department of Military Psychology, Fourth Military Medical University, Xi’an, China; ^3^State Key Laboratory of Military Stomatology and National Clinical Research Center for Oral Diseases and Shaanxi Engineering Research, Department of Anethesiology, Center for Dental Materials and Advanced Manufacture, School of Stomatology, Fourth Military Medical University, Xi’an, China; ^4^Department of Anatomy, School of Basic Medicine, Fourth Military Medical University, Xi’an, China

**Keywords:** Shank3b, social behavior, anterior cingulate cortex, synapse, glycogen synthase kinase 3β

## Abstract

Social deficiency is one of the core syndromes of autism spectrum disorders (ASD), for which the underlying developmental mechanism still remains elusive. Anterior cingulate cortex (ACC) plays a key role in integrating social information and regulating social behavior. Recent studies have indicated that synaptic dysfunction in ACC is essential for ASD social defects. In the present study, we investigated the development of synapses and the roles of glycogen synthase kinase 3β (GSK-3β), which mediates multiple synaptic signaling pathways in ACC by using *Shank3b*^−/−^ mice (a widely used ASD mouse model). Our data revealed that *Shank3b* mutation abolished the social induced c-Fos expression in ACC. From 4 weeks post-birth, neurons in *Shank3b*^−/−^ ACC exhibited an obvious decrease in spine density and stubby spines. The length and thickness of post-synaptic density (PSD), the expression of vesicular glutamate transporter 2 (vGlut2) and glutamate receptor 2 (GluR2), and the frequency of miniature excitatory post-synaptic currents (mEPSCs) were significantly reduced in *Shank3b*^−/−^ACC. Interestingly, the levels of phosphorylated GSK-3β (Ser9), which inhibits the activity of GSK-3β, decreased along the same time course as the levels of GluR2 increased in ACC during development. *Shank3b* mutation leads to a dramatic increase of pGSK-3β (Ser9), and decrease of pPSD95 (a substrate of GSK-3β) and GluR2. Local delivery of AAV expressing constitutively active GSK-3β restored the expression of GluR2, increased the spine density and the number of mature spines. More importantly, active GSK-3β significantly promoted the social activity of *Shank3b*^−/−^ mice. These data, in together, indicate that decrease of GSK-3β activity in ACC may contribute to the synaptic and social defects of *Shank3b*^−/−^ mice. Enhancing GSK-3β activity may be utilized to treat ASD in the future.

## Introduction

Autism spectrum disorders (ASDs) are a group of neural developmental disorders characterized by repetitive stereotype behaviors and social defects (Takumi et al., 2019). Hundreds of related genetic mutations have been identified in human patients (Jacob et al., 2019). Among these candidate genes, Src-homology domain 3 (SH3) and multiple ankyrin repeat domains 3b (*Shank3b*) is one of the few genes which can cause the core syndrome of ASD at single mutation (Peca et al., 2011; Varghese et al., 2017). SHANK3 is a post-synaptic scaffold protein widely expressed by excitatory neurons, and interacts with multiple synaptic proteins (Monteiro and Feng, 2017). Even heterozygous *Shank3b* mice exhibit repetitive grooming and social defects (Dhamne et al., 2017; Qin et al., 2018). Previous studies have revealed that dysfunction of striatum glutamatergic transmission is essential for the repetitive grooming behavior of *Shank3b*^−/−^ mice (Wang et al., 2017). The mechanism underlying the social deficiency of *Shank3b*^−/−^ mice remains largely unknown.

Proper social behavior is essential for mammalian survival and reproduction. Among the brain regions involved in the regulation or generation of social behavior, anterior cingulate cortex (ACC) is regarded as the core region for integrating social information afferents from other brain regions such as the hippocampus, thalamus, and prefrontal cortex (PFC; Apps et al., 2016). Recently, our study demonstrated that the dysfunction of ACC, especially the dysfunction of excitatory synaptic transmission, accounted for the social deficiency in *Shank3b*^−/−^ mice (Guo et al., 2019). Developmental synaptic deficiency has been proposed to be a key pathological change of ASDs (Lima Caldeira et al., 2019). How *Shank3b* mutation leads to synaptic defects in ACC is an interesting topic to be explored.

Glycogen synthase kinase 3β (GSK-3β) is a conserved serine/threonine kinase highly abundant in the brain. It is involved in multiple cellular process and signaling pathways, particularly Wnt signaling and mTOR signaling (Meffre et al., 2014; Hermida et al., 2017). In neurons, the function of GSK-3β is closely related to synaptic development and plasticity (Hur and Zhou, 2010). It can phosphorylate the N-methyl-D-aspartate (NMDA) receptor and post-synaptic density protein 95 (PSD95), thereby modulating the function of glutamic synapses (Peineau et al., 2007; Nelson et al., 2013). Considering that SHANK3B is located mainly in the post-synaptic components of excitatory synapses, we are curious as to whether GSK-3β are involved in the social deficiency of *Shank3b*^−/−^ mouse.

In the present study, we investigated the synaptic defects in the ACC of *Shank3b*^−/−^ mice during development. Our data demonstrated that the decrease of GSK-3β activity in ACC contributed to the synaptic and social deficiency of *Shank3b*^−/−^ mice.

## Materials and Methods

### Animals and Virus Injection

*Shank3b*^−/−^ mice were a gift from Prof. Guoping Feng as described (Peca et al., 2011). The mice were maintained and housed in the animal facility of the Fourth Military Medical University at an ambient temperature of 25°C, with a 12 h light and 12 h dark cycle and bred by mating heterozygous male with heterozygous female. The mice were given ad libitum access to food and water. All procedures conducted with mice were in compliance with the guidelines for experimental animal care and use by the Committee of the Animal Care and Use Committee of Fourth Military Medical University. The protocol of animal experiments was approved by the Committee of the Animal Care and Use Committee of Fourth Military Medical University.

Recombinant adeno-associated virus (rAAV) expressing constitutive active GSK-3β (converting Ser9 into Ala9, 1 × 10^13^ PFU) driven by hSyn promoter was obtained from BrainVTA (Wuhan, China). The virus was injected into the bilateral ACC of *Shank3b*^−/−^ mice (4 weeks old) at 0.2 mm right or left to Bregma, 0.75 mm anterior to Bregma with 400 nl per point and 1 mm in depth. AAV expressing luciferase was used as control.

### Golgi Staining

Golgi staining was performed as previously described with minor modifications (Zhang et al., 2011). Animals were perfused with 0.9% saline solution or 0.01 M phosphate-buffered saline (PBS; pH 7.4). The brain tissue was removed and immersed in Golgi-cox solution (consisting 5% potassium chromate, 5% potassium dichromate, and 5% mercuric chloride) for further fixation, after which the tissue was maintained in the dark at room temperature for 2–3 days. Then, the brains were transferred to a fresh Golgi-Cox Solution for additional 14 days. After that, the brains were transferred to 25%–30% sucrose for 2 more days. Coronal sections (100–200 μm) were cut serially. For staining, brain sections were washed in deionized water for 1 min, placed in 50% NH_4_OH for 30 min, and subsequently in fixing solution (Kodak; Rochester, NY, USA) for an additional 30 min. The sections were then incubated in 5% sodium thiosulfate for 10 min. After rinsing with distilled water, dehydration with gradient ethanol was performed. The sections were finally mounted and observed under the bright field of confocal microscope FV1000, images were taken by z-stack scanning with the excitation wavelength of 405 nm, and then the virtual color was converted into red color.

### Western-Blotting

ACC tissues were carefully dissected and lysed by an RIPA buffer at the presence of a proteinase inhibitors cocktail. Protein concentration was determined by BCA assay. Protein samples were separated in 10%–12% acrylamide gels by SDS-PAGE and transferred to PVDF membranes. Membranes were blocked in TBS containing 0.1% (v/v) Tween 20 (TBS-T) and 5% (w/v) nonfat milk before incubation with primary antibodies. The following antibodies were used: rabbit anti-p-GSK-3β (ser9; 1:1,000; 9323s, Cell Signaling Technology), rabbit anti-total GSK- 3β (1:1,000; ab93926, Abcam), rabbit anti-β-catenin (1:1,000; #8480, Cell Signaling Technology), rabbit anti-p70-S6 (Thr421/Ser424; 1:1,000; #9204, Cell Signaling Technology), rabbit anti-mTOR (1:1,000; 2971s, Cell Signaling Technology), rabbit anti-GluR2 (1:1,000; 11994-1-AP, ProteinTech), rabbit anti-pPSD95 (1:1,000; ab172628, Abcam), and rabbit anti-vGlut2 (1:1,000; #71555, CST). After four washes with TBS-T, membranes were incubated with HRP-conjugated anti-mouse secondary antibodies (Cat. CW0102S, CWBIO Company Limited), or HRP-conjugated anti-rabbit secondary antibodies (Cat. EK020; 1:3,000; Zhuangzhi Biotech Company Limited). Bands were visualized by an ECL kit (Thermo). Images were taken by Tanon imaging system, and analyzed by ImageJ. For quantification of blots, the ratios of $\frac{\text{gray scale of target protein}}{\text{gray scale of}\beta \text{-actin}}$ in experimental groups were compared to those of control groups.

### Behavior Assay

#### Three-Chamber Test

The 3-Chamber apparatus was an opaque acrylic box with two pull-out doors and three chambers. Each chamber was identical in size (41 × 20 cm), with the dimensions of the entire box being 63 (length) 43 (width) × 23 cm (height). There was a 10-cm gap between adjacent chambers which could be opened or closed with the removable doors. Before tests, mice were individually habituated in the 3-Chamber apparatus for 10 min. After habituation, a C57 stimulus mouse of same age and same sex was placed in the inverted wired cylinder in the “social chamber.” The cylinder in the “non-social chamber” remained empty. The time the tested mice spent in the social vs. non-social chambers during the 10 min test period was measured. Only when all four paws entered the chamber, the mouse was considered to be within a specific chamber. The behaviors of each mouse were video-recorded during the entire test to assess the details of social behavior (rear, contact, sniff, grooming, stretch, withdrawal, and nose-to-nose). The chamber was cleaned by 75% ethanol between each test. The time and traveled distance were analyzed by using SMART3.0 software (Panlab Harvard Apparatus, Spain).

#### Resident-Juvenile-Intruder Home-Cage Test

Social interaction was examined as described with minor modifications (Felix-Ortiz and Tye, 2014). Briefly, a male adult *Shank3b*^−/−^ mouse was allowed to explore freely for 1 min (habituation) in his home cage. Another novel juvenile (3–4 weeks old) male C57BL/6 mouse was introduced to the cage and allowed to explore freely for 3 min (test session). Juvenile mice were used to avoid mutual aggression. All behaviors were video recorded and analyzed by a researcher blind to the testing condition. The time and frequency of direct contact (pushing the snout or head underneath the juvenile’s body and crawling over or under the juvenile’s body) were measured.

### Immunohistochemistry

Animals were sacrificed and perfused intracardially with 4% cold paraformaldehyde phosphate buffer (pH 7.4). Brain tissue was cryoprotected by 20%–30% sucrose. For each mouse, serial sections (20 μm in thickness for each section) were cut and all the sections were collected onto eight slides. For immunostaining, the sections were blocked by 0.01 M PBS containing 0.3% Triton X-100 and 3% bovine serum albumin (BSA) for 1 h. Primary antibodies were used as following: rabbit anti-c-Fos (1:500; F7799, Sigma), mouse anti-NeuN (1:600; ab104224, Abcam), guinea pig anti-vGluT2 (1:200; 135404, synaptic system), mouse anti-MAP2 (1:400; MAB3418X, Millipore), and rabbit anti-vGlut2 (1:1,000; #71555, CST). After primary antibodies incubation and washing with PBS, sections were incubated with their corresponding secondary antibodies conjugated with Alexa Fluor 594 or Alexa Fluor 488 (Jackson Immunoresearch) for 2–4 h at room temperature protected from light. The nuclei were counterstained by DAPI (1:1,000, Sigma). All immunostained sections were photographed under a confocal microscope (FV1000, Olympus) with the same setting.

### Electron Microscopic Study

Animals were perfusion fixed with a mixture of 4% paraformaldehyde containing 1% glutaraldehyde. Tissue sections of 50 μm were prepared with a vibratome and further fixed with 1% osmium tetroxide, dehydrated with graded ethanol, replaced with propylene oxide, and flat-embedded in Epon 812. The sections were trimmed under a stereomicroscope and mounted onto blank resin stubs for ultrathin sectioning. Ultrathin sections (70–90 nm) were prepared on an LKB Nova Ultratome (Bromma). After being counterstained with uranyl acetate and lead citrate, the sections were examined under a JEM-1230 electron microscope (JEM, Tokyo).

### Patch-Clamp Recording

Coronal brain slices (300 μm) at the level of the ACC were prepared. Slices were transferred to submerged recovery chamber with oxygenated (95% O_2_ and 5% CO_2_) artificial CSF containing (in mM) 124 NaCl, 2.5 KCl, 2 CaCl_2_, 1 MgSO_4_, 25 NaHCO_3_, 1 NaH_2_PO_4_, and 10 glucose at room temperature for at least 1 h. The recording pipettes (3–5Ω) were filled with a solution containing (in mM) 145 K-gluconate, 5 NaCl, 1 MgCl_2_, 0.2 EGTA, 10 HEPES, 2 Mg-ATP, 0.1 Na_3_-GTP, and 10 phosphocreatine disodium (adjusted to pH 7.2 with KOH). The internal solution (in mM) 140 cesium methanesulfonate, 5 NaCl, 0.5 EGTA, 10 HEPES, 2 MgATP, 0.1 Na_3_GTP, 0.1 spermine, 2 QX-314 bromide, and 10 phosphocreatine disodium (adjusted to pH 7.2 with CsOH) was used in the experiment. For miniature EPSC miniature excitatory post-synaptic current (mEPSC) recording, 1 μM TTX and 100 μM picrotoxin were added in the perfusion solution. Picrotoxin (100 μM) was present to block GABAA receptor-mediated inhibitory synaptic currents. Data were excluded when the resting membrane potential of neurons was more positive than −60 mV and action potentials did not have overshoot. Data were filtered at 1 kHz, and digitized at 10 kHz. The mEPSCs were detected and analyzed using Mini Analysis (Synaptosoft Inc., Decatur, GA, USA).

### Morphological Analysis

All images of Golgi staining and immunofluorescent staining were taken by Olympus FV1000. For sholl analysis, in cases where individual neurons could not be identified, branches of non-target neurons were manually erased so that the target neuron with full processes was clearly left in the field. The branch intersections and length of dendrites were determined by serial circles surrounding cell body every 5 μm until reaching the end of the longest dendrite using ImageJ. For spine analysis, IMARIS 7.5 filament tracer was used to reconstruct each dendrite. Using customized algorithms, spines were classified as filopodia, stubby, long-thin, or mushroom (Berry and Nedivi, 2017). For the fluorescent intensity, images were taken under the same setting (at least five images from each animal) and analyzed by ImageJ as described (Bhat et al., 2017).

### Data Analysis

Western-blotting images were analyzed by ImageJ. For immunohistochemistry and Western-blots, at least three biological repeats were performed for each experiment. For behavior study, at least 15 mice were included in each group. The data were presented as means ± standard error of mean (SEM). The normality was assessed by the Shapiro–Wilk test. Data were analyzed by one-way analysis of variance (ANOVA) except for Sholl analysis which was analyzed by two-way ANOVA, followed by Dunnett *post hoc* using SPSS l6.0 (Chicago, IL, USA) or by unpaired, two-tailed Student’s *t*-test. *P*-values less than 0.05 were considered as statistically significant.

## Results

### Compromised Response of ACC Neurons to Social Stimulus in *Shank3b*^−/−^ Mice

**Figure 1:**
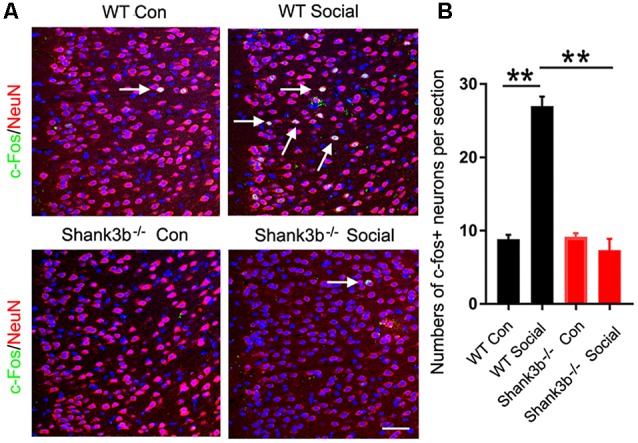
Response of anterior cingulate cortex (ACC) neurons to social stimulation in WT and *Shank3b*^−/−^ mice.** (A)** Double-immunostaining of c-Fos/NeuN in the ACC of WT control (Con) mice, social stimulated WT mice (WT social), *Shank3b*^−/−^ control mice and social stimulated *Shank3b*^−/−^ mice. **(B)** Quantification of c-Fos/NeuN-positive cells. Notice that social behavior elicits c-Fos expression in WT ACC but not in *Shank3b*^−/−^ ACC. Arrows point to double-positive cells. Bar = 120 μm. Values represent mean ± SEM. **P < 0.01. *N* = 5 mice per group. One-way analysis of variance (ANOVA).

We first analyzed the response of ACC neurons to social stimulation. The expression of c-Fos, a quick-response gene widely used to reflect neuronal activation, was examined at 1 h after the 3-chamber test. The testing mice were placed into the “social chamber” for 10 min, and the control mice placed in the “non-social chamber” for 10 min. The average social time was approximately 270 s. Very few or no c-Fos-positive neurons could be observed in the ACC of both WT and *Shank3b*^−/−^ mice under control conditions (Figure 1A, left panels; Figure 1B). A lot of c-Fos-positive neurons were found in the ACC of social stimulated WT mice, while the number of c-Fos-positive neurons in the ACC of social stimulated *Shank3b*^−/−^ mice remained at a similar level as that in WT control (Figure 1A, right panels; Figure 1B). Both in WT and *Shank3b*^−/−^ mice, there was no c-Fos expression in striatum, the key region involved in the grooming phenotype of *Shank3b*^−/−^ mice (Supplementary Figure S1). These data indicated that ACC, but not striatum, responds to social stimulation, which can be compromised by *Shank3b* mutation.

### Abnormal Synaptic Development in the ACC of *Shank3b*^−/−^ Mice

**Figure 2:**
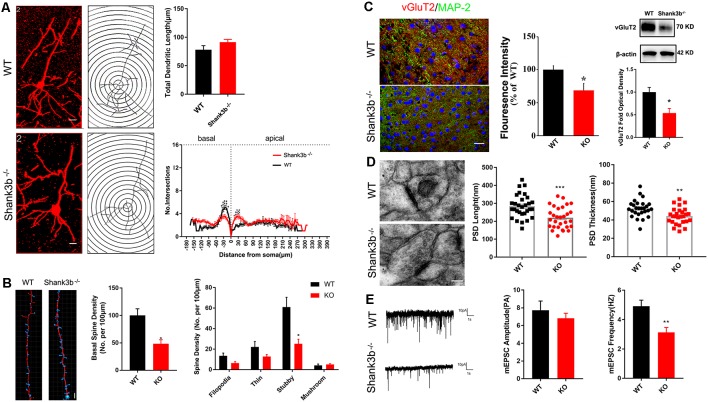
Effects of *Shank3b* mutation on the dendrite and synapse development in ACC.** (A)** Sholl analysis of Golgi images of pyramidal neurons in WT and *Shank3b*^−/−^ ACC 4 weeks post-birth. Notice the fewer branches in the basal dendrites of *Shank3b*^−/−^ mice of 4 weeks old. **(B)** Imaris analysis of spines in basal dendrites of WT and *Shank3b*^−/−^ neurons. Notice the decrease of total spine density and stubby spines. **(C)** Double-immunostaining of vesicular glutamate transporter 2 (vGlut2)/MAP2 and Western-blotting of vGluT2. Notice the weaker expression of vGluT2 in *Shank3b*^−/−^ ACC. **(D)** Electron microscopic study of synaptic ultrastructure in WT and *Shank3b*^−/−^ ACC at 4 weeks post-birth. The average length and thickness of post-synaptic density (PSD) was reduced. **(E)** Patch-clamp recording of miniature excitatory post-synaptic current (mEPSC) in WT and *Shank3b*^−/−^ ACC. Notice the reduction of frequency of mEPSC in *Shank3b*^−/−^ ACC. Bars = 2 μm **(A,B)**, 150 μm **(C)** and 135 nm **(D)**. Values represent mean ± SEM. *P < 0.05. **P < 0.01. *N* = 6 mice per group in **(A,B)**, 30 neurons per group in **(C)**, five mice per group in **(D)** and six neurons per group **(E)**. Two-way ANOVA **(A)**, One-way ANOVA **(B)** and Student’s *t*-test **(C–E)**.

Because SHANK3B is mainly expressed in synaptic sites of excitatory neurons, we focused on the synaptic development of pyramidal neurons in ACC. Golgi staining was conducted at 2, 3, and 4 weeks post-birth. A gradual morphological differentiation was observed. Typical spines along dendrites were not detected until 4 weeks post-birth (Supplementary Figure S2). Therefore, in the present study, we mainly analyzed phenotype at 4 weeks post-birth. Sholl analysis showed that there was no difference in the total dendritic length between *Shank3b*^−/−^ ACC neurons and WT ACC neurons. However, significantly less basal dendrite branches and more proximal apical branches were found in *Shank3b*^−/−^ ACC neurons (Figure 2A), indicating that *Shank3b* may influence the dendrite development of ACC pyramidal neurons.

We next evaluated the spine development in the ACC neurons of *Shank3b*^−/−^ mice with focus on basal dendrites. Quantification showed that the density of spines along basal dendrites was remarkably reduced in *Shank3b*^−/−^ neurons as compared with that of WT neurons (Figure 2B, left and middle panels). Further analysis of the sub-types of spines showed that there was no change of immature filodopia and thin spines. However, the number of stubby spines decreased significantly (Figure 2B, right panel), indicating that the maturation of excitatory synapses may be affected by *Shank3b* mutation. In line with this result, immunohistochemistry and Western-blotting showed that the expression of vesicular glutamate transporter 2 (vGlut2) was significantly reduced in the ACC of *Shank3b*^−/−^ mice (Figure 2C). Electron microscopic study further revealed that the average length and thickness of PSD were significantly reduced in the ACC of *Shank3b*^−/−^ mice (Figure 2D). Further, patch-clamp recording showed that the frequency of mEPSC was significantly reduced in the ACC of *Shank3b*^−/−^ mice (Figure 2E). These data indicate that *Shank3b* mutation may impair the formation and function of excitatory synapses in ACC.

### Decrease of GSK-3β Activity and GluR2 Expression in the ACC of *Shank3b*^−/−^ Mice

**Figure 3:**
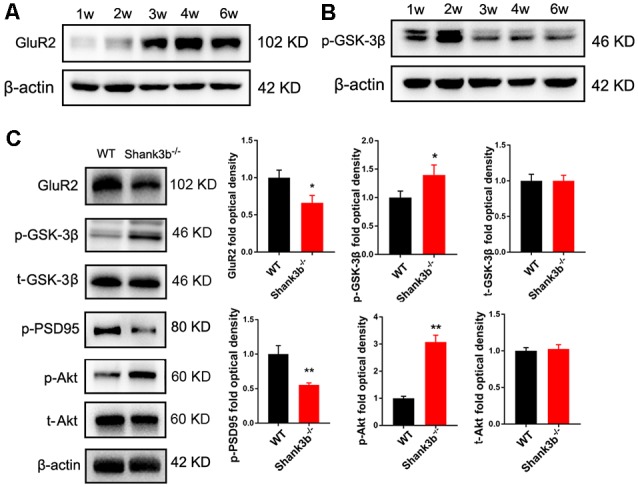
Expression of glutamate receptor 2 (GluR2) and glycogen synthase kinase 3β (GSK-3β) during postnatal development and in *Shank3b*^−/−^ ACC.** (A)** Western-blotting of GluR2 in the ACC of 1, 2, 3, 4 and 6 weeks (w) old mice. **(B)** Western-blotting of p-GSK-3β (S9) in the ACC of 1, 2, 3, 4 and 6 w old mice. Notice the increase of GluR2 and decrease of p-GSK-3β (S9) from 3 w on. **(C)** Western-blotting of GluR2, p-GSK-3β, total GSK-3β (t-GSK-3β), phosphorylated PSD-95 (pPSD-95), phosphorylated Akt (p-Akt), and total Akt (t-Akt) in WT and *Shank3b*^−/−^ ACC. Notice the up-regulation of p-GSK-3β and p-Akt, and down-regulation of GluR2 and pPSD-95 in *Shank3b*^−/−^ ACC. Values represent mean ± SEM. *P < 0.05. **P < 0.01. *N* = 6 mice per group. Student’s *t*-test.

To explore the possible underpin mechanisms, we focused on GSK-3β signaling, a key kinase involved in the dendrites growth and polarization of neurons. GSK-3β interacts with multiple signaling pathways, such as Wnt/β-catenin signaling, mTOR signaling and MAPK signaling (Hur and Zhou, 2010; Hermida et al., 2017), and phosphorylates many cytoskeleton proteins (Buttrick and Wakefield, 2008), thereby regulating dendrite development. We first investigated the expression of phosphorylated GSK-3β (p-GSK-3β, Ser 9), an inactive form of GSK-3β, and glutamate receptor 2 (GluR2), a major AMPA receptor which is correlated to synaptic localization of SHANK3 (Grimes and Jope, 2001; Ha et al., 2018). During postnatal development of ACC, the expression of GluR2 increased from 3 weeks, and remained at a high level from then on (Figure 3A). Interestingly, the expression of p-GSK-3β (Ser 9) in ACC dramatically decreased from 3 weeks, and stayed at a low level from then on (Figure 3B). These data revealed an inverse expression trend of GSK-3β activity and GluR2 protein during development. We then analyzed the expression of GSK-3β and GluR2 in the ACC of *Shank3b*^−/−^ mice at 4 weeks (Figure 3C). The expression of GluR2 decreased significantly in *Shank3b*^−/−^ ACC. The levels of p-GSK-3β (Ser 9) were significantly increased in the ACC of *Shank3b*^−/−^ mice, while the levels of total GSK-3β kept unchanged (Figure 3C). Since p-GSK-3β (Ser 9) is an inhibitory form of GSK-3β, we next examined the phosphorylation of PSD95, a substrate of GSK-3β. The results showed that p-PSD95 was significantly reduced in *Shank3b*^−/−^ ACC (Figure 3C). Further, we assessed the expression of Akt and p70-S6, two key proteins which modulate GSK-3β activity. The phosphorylation of Akt was increased in the ACC of *Shank3b*^−/−^ mice while the expression of total Akt and p70-S6 remained unchanged (Figure 3C; Supplementary Figure S3A). The levels of p-GSK-3β in mPFC and striatum of *Shank3b*^−/−^ mice remained at similar levels as those in WT mice (Supplementary Figure S3B). These data indicated that GSK-3β activity is lowered in *Shank3b* deficient neurons.

### Rescue of Synaptic Development and Social Behavior by ACC Expression of Constitutive Active GSK-3β

**Figure 4:**
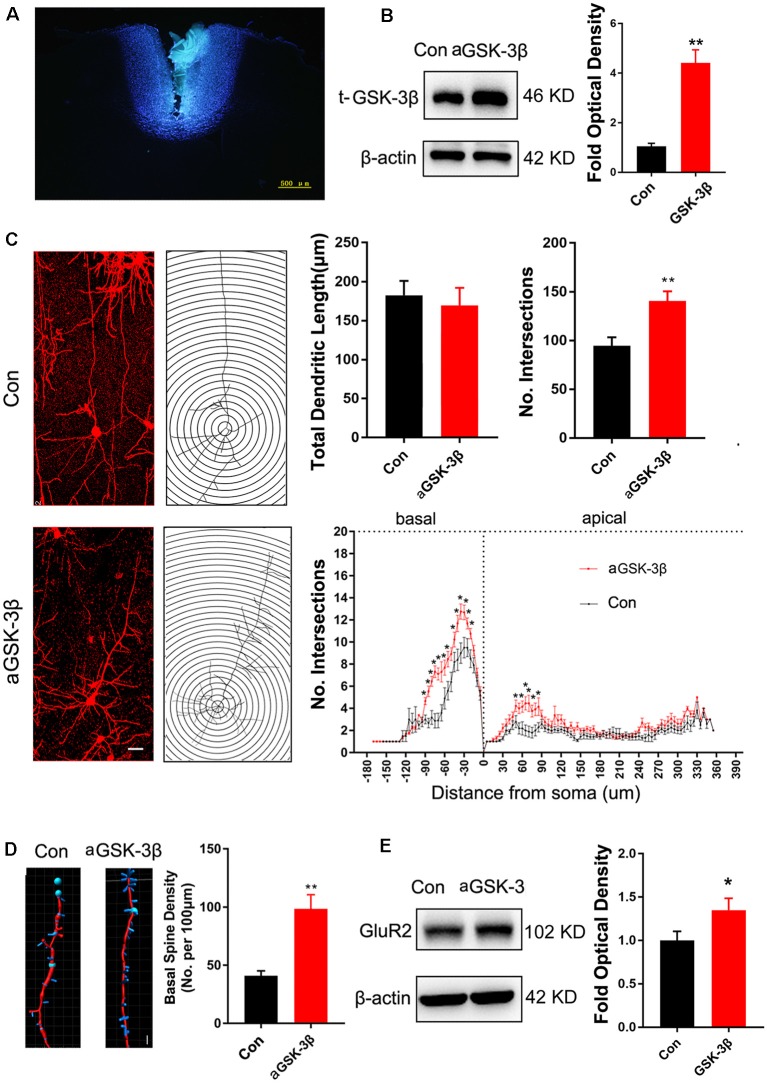
Effects of over-expressing constitutive active GSK-3β (aGSK-3β) in *Shank3b*^−/−^ ACC on the development of dendrites and spines.** (A)** Hoescht33342 staining of injection site at 24 h after virus injection in *Shank3b*^−/−^ mice of 4 weeks old. **(B)** Western-blotting of t-GSK-3β in ACC at 3 weeks after injecting AAV expressing aGSK-3β. **(C)** Sholl analysis of Golgi images of pyramidal neurons in control and aGSK-3β treated *Shank3b*^−/−^ ACC. Notice the increase of dendritic intersections, and particularly basal branches in the aGSK-3β treated *Shank3b*^−/−^ neurons. **(D)** Imaris analysis of spines along basal dendrites of control and aGSK-3β treated *Shank3b*^−/−^ neurons. Notice the increase of spine density by aGSK-3β. Values represent mean ± SEM. *P < 0.05. **P < 0.01. Bar = 20 μm **(C)** and 2 μm **(D)**. *N* = 3 mice per group **(B)**, and 6 mice per group **(C–E)**. Student’s *t*-test **(A,D,E)**. Two-way ANOVA **(B)**.

To investigate the effects of enhancing GSK-3β activity on synapse development, we injected AAV-expressing constitutive active GSK-3β (aGSK-3β) into the ACC of *Shank3b*^−/−^ mice. Our preliminary experiments showed that it was very hard to precisely inject the virus into ACC at 3 weeks or earlier, possibly due to the small size of ACC (data not shown). We performed virus injection at 4 weeks post-birth and the area of virus diffusion was determined by mixing Hoeschst33342 with virus aliquots (Figure 4A). Three weeks later, significant up-regulation of total GSK-3β was confirmed by Western-blotting (Figure 4B). Although the total length of dendrites was not significantly affected by aGSK-3β, more dendrite intersections were found in *Shank3b*^−/−^ ACC treated with aGSK-3β than control (Figure 4C). Further, Sholl analysis showed that branches along both the basal and apical dendrites were significantly increased in aGSK-3β-treated neurons (Figure 4C). More importantly, not only was the spine density increased (Figure 4D), but the mature spines (stubby and mushroom spines) were also remarkably increased (Supplementary Figure S4). In addition, the expression of GluR2 was significantly up-regulated in the *Shank3b*^−/−^ ACC treated with aGSK-3β (Figure 4E). These data indicated that aGSK-3β could rescue the synapse formation in *Shank3b* deficient ACC.

**Figure 5:**
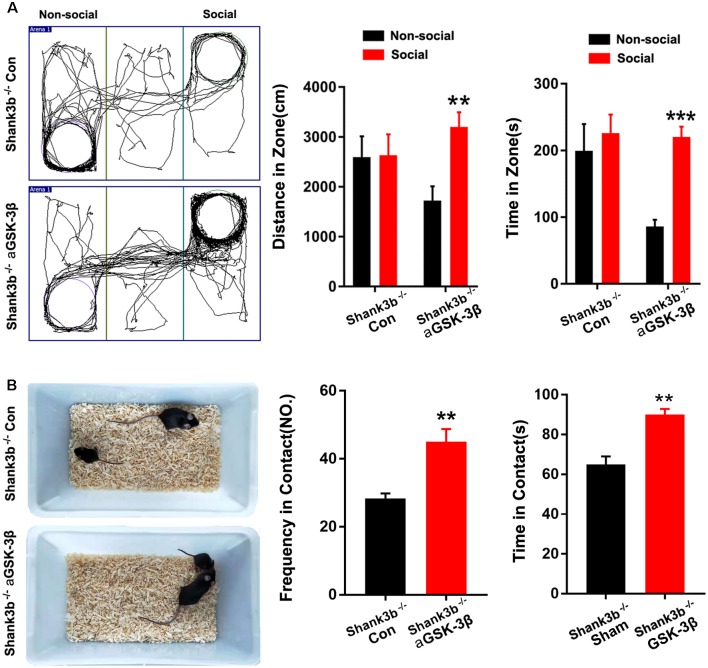
Effects of over-expressing aGSK-3β in *Shank3b*^−/−^ ACC on social behavior. **(A)** Three-chamber tests of control *Shank3b*^−/−^ mice and *Shank3b*^−/−^ mice treated with aGSK-3β at 3 weeks after virus injection. Notice that aGSK-3β treatment significantly rescued the social preference of *Shank3b*^−/−^ mice. **(B)** Resident-juvenile-intruder home-cage test of control *Shank3b*^−/−^ mice and *Shank3b*^−/−^ mice treated with aGSK-3β. Notice that aGSK-3β treatment significantly enhanced social contact of *Shank3b*^−/−^ mice with juvenile intruder. Values represent mean ± SEM. **P < 0.01. ***P < 0.001. *N* = 15 mice per group **(A,B)**. One-way ANOVA **(A)**. Student’s *t*-test** (B)**.

We next tested whether over-expressing aGSK-3β in ACC could affect the social behavior of *Shank3b*^−/−^ mice. In the 3-chamber test which measures social approach and preference for social novelty, control *Shank3b*^−/−^ mice spent similar time exploring the cylinder containing social mice as exploring the empty cylinder (Figure 5A). *Shank3b*^−/−^ mice treated with aGSK-3β spent obviously more time in the box containing social mice (Figure 5A). In the resident-juvenile-intruder home-cage test which measures social interaction, *Shank3b*^−/−^ mice treated with aGSK-3β showed significantly more direct contact with the juvenile intruder, while *Shank3b*^−/−^ mice treated with control virus showed no obvious interests to the juvenile intruder (Figure 5B). These data suggested that over-expressing aGSK-3β in ACC can rescue the social deficiency of *Shank3b*^−/−^ mice.

## Discussion

Recently, we reported that ACC is a key region for the social deficit of *Shank3b*^−/−^ mice. In the present study, we further analyzed the effects of *Shank3b* mutation on the morphological development of ACC neurons with focus on synaptogenesis. Our data revealed a defect of excitatory synaptic development and a decrease of GSK-3β activity in the ACC of *Shank3b*^−/−^ mice. Interestingly, the expression of constitutive active GSK-3β in ACC rescued both the synaptic and social deficiency caused by *Shank3b* mutation. Our data further supported a crucial role of ACC in social function. Considering the extensive connection of ACC, it will be interesting to investigate how the interaction between ACC and its connecting regions, such as PFC, affects social behavior.

Of the current hypothesis about the development of ASD, “developmental synaptopathie” has drawn more and more attention in recent years (Ebrahimi-Fakhari and Sahin, 2015), which implies that the disruption of the synaptic excitation and inhibition (E/I) is crucial for the progress of ASD (Howell and Smith, 2019). Shank3 is one of the few genes whose single mutation is sufficient to induce typical autism syndrome. The dysfunction of excitatory synapses in the striatum of *Shank3*^−/−^ mice has been attributed to the reduction of NMDA/AMPA ratio which in turn results in the silence of excitatory synapses (Jaramillo et al., 2016). Our data showed that in the ACC of *Shank3*^−/−^ mice, the density of spines, the length and thickness of PSD, and the expression of GluR2 were significantly reduced. These data demonstrated the abnormal development of excitatory synapses in the ACC of *Shank3b*^−/−^ mice. The reduction of vGluT2 is interesting. Our electrophysiological data are in line with this observation. It is possible that post-synaptic SHANK3B may affect synaptic transmission through neurexin-neuroligin-mediated transsynaptic signaling (Arons et al., 2012). Considering that previous studies have reported presynaptic localization of Shank3 in developing neurons (Halbedl et al., 2016; Wu et al., 2017) and that our data were obtained at 4w post-birth, it may be possible that SHANK3 may play a role in the presynaptic function during development. Because SHANK3B could also be expressed by GABAergic neurons, the possibility that abnormal inhibition may affect the morphology of neurons and regulate the structural and functional plasticity of glutamatergic synapses is interesting, and deserves to be further investigated.

The existence of six protein-protein interaction domains in SHANK3B imparts it with the ability to interact with or affect diverse synaptic proteins. Dozens of Shank3-interacting proteins have been identified, such as Contactin, Homer, and SAPAP (Monteiro and Feng, 2017). Recently, SHANK3B has been reported to directly interact with β-catenin (Qin et al., 2018), a Wnt signaling protein which can modulate synapse formation and stability. As an upstream molecule of β-catenin in Wnt signaling, GSK-3β is mainly distributed in post-synaptic components. Our data showed a significant enhancement of p-GSK-3β (S9) in the ACC of *Shank3b*^−/−^ mice, suggesting that GSK-3β activity was inhibited in *Shank3b*^−/−^ mice, which is consistent with previous reports that Wnt/β-catenin is activated in *Shank3b* deficient PFC (Qin et al., 2018). The inverse expression trend of GSK-3β and GluR2, and the rescue effects of aGSK-3β on GluR2 indicated a relationship between the expression of GluR2 and GSK-3β activity. The involvement of GSK-3β in the synapse dysfunction of other ASD models (e.g., FMR1^−/−^ mice) has been documented (Mines et al., 2010; Caracci et al., 2016). Knocking out *GSK-3β* in neurons resulted in fewer spines (Kondratiuk et al., 2017), similar to what we have observed. Whether SHANK3B could directly interact with GSK-3β is worthy of further exploration.

Importantly, our data showed that expressing constitutive active GSK-3β in *Shank3b*^−/−^ ACC could rescue both synaptic development and social function. The rescue of GluR2 expression may be achieved by its downstream β-catenin signaling. So far, there still lacks effective treatment for the social deficiency in ASD patients. Considering too much activation of GSK-3β by constitutive a GSK-3β may lead to inhibitory effects, pharmacologically enhancing GSK-3β activity might be an alternative for the improving social behavior in ASD patients.

Considering the multiple signaling GSK-3β involved and the wide distribution of GSK-3β, it is possible that this synaptic function of SHANK3B may also be important for the pathology of many other neuropsychiatric and neurodevelopmental disorders, such as Alzheimer’s disease and Schizophrenia.

## Data Availability Statement

The datasets generated for this study are available on request to the corresponding author.

## Ethics Statement

The animal study was reviewed and approved by the Committee of the Animal Care and Use Committee of Fourth Military Medical University. Written informed consent was obtained from the owners for the participation of their animals in this study.

## Author Contributions

MW, XL and YH conducted most of the experiments, collected and analyzed the data. HZ and YZ contributed to morphological analysis. JK contributed to electron microscropic study. FW contributed to electrophysiology. JC contributed to Golgi staining. XL contributed to behavior analysis. SW and YW conceived the study, provided financial support, analyzed the data, and prepared the manuscript.

## Conflict of Interest

The authors declare that the research was conducted in the absence of any commercial or financial relationships that could be construed as a potential conflict of interest.

## Supplementary Material

FIGURE S1 Double-immunostaining of c-Fos with NeuN in the striatum of WT and *Shank3b*^−/−^ mice after social stimulation. Notice that there are no c-Fos-positive cells in the striatum of WT and *Shank3b*^−/−^ mice after social stimulation.

FIGURE S2 Images of Golgi staining in ACC at 2w **(A)**, 3w **(C)** and 4w **(E)** post-birth. Images of dendritic spines in pyramidal neurons at 2w **(B)**, 3w **(D)** and 4w **(F)** post-birth 4.

FIGURE S3 **(A)** Western blotting and quantification of p70-S6 in WT ACC and *Shank3b*^−/−^ ACC. *N* = 6 mice per group. **(B)** Western-blotting and quantification of p-GSK-3βin the mPFC and striatum of WT and *Shank3b*^−/−^ mice. *N* = 4 mice per group.

FIGURE S4 Densities of different types of basal spines of control and aGSK-3β treated *Shank3b*^−/−^ ACC. Notice the increase of stubby and mushroom spines by aGSK-3β. Values represent mean ± SE. **P* < 0.05, ****P* < 0.001.
