# Supplementary figures and images for: Decrease of GSK-3β Activity in the Anterior Cingulate Cortex of Shank3b−/− Mice Contributes to Synaptic and Social Deficiency
Source: Front Cell Neurosci. 2019 Oct 23;13:447. doi: 10.3389/fncel.2019.00447 (PMC6843030; doi:10.3389/fncel.2019.00447)

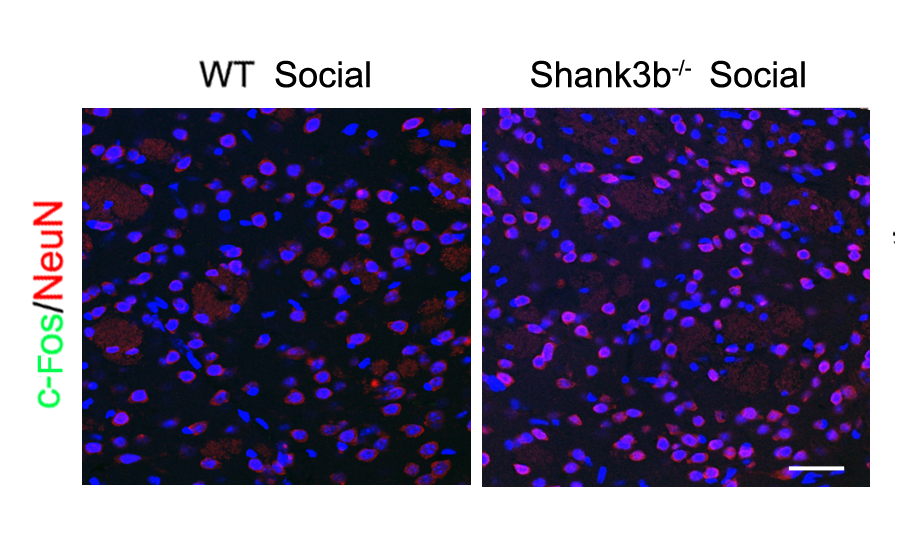

Supplement: FIGURE S1 — Double-immunostaining of c-Fos with NeuN in the striatum of WT and Shank3b−/− mice after social stimulation. Notice that there are no c-Fos-positive cells in the striatum of WT and Shank3b−/− mice after social stimulation. [file Image_1.tif]

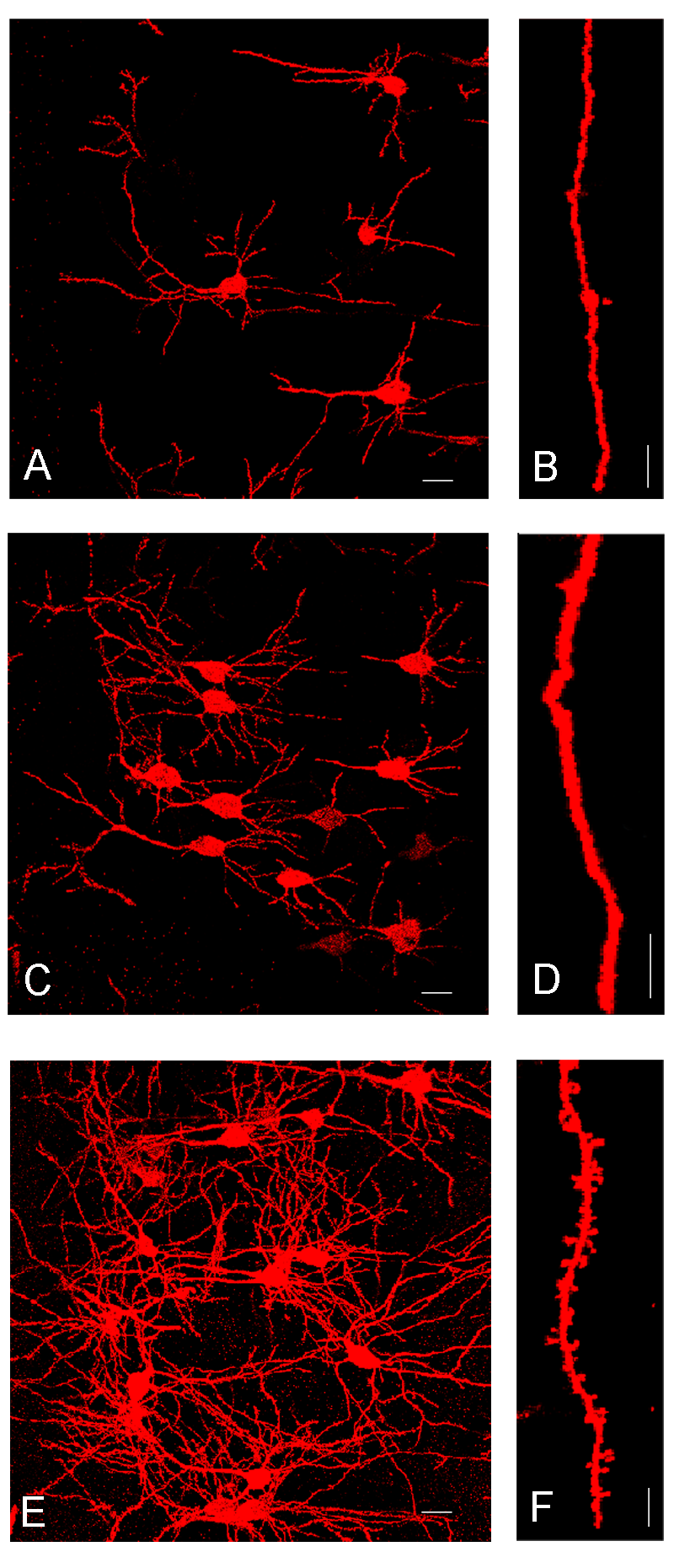

Supplement: FIGURE S2 — Images of Golgi staining in ACC at 2w (A), 3w (C) and 4w (E) post-birth. Images of dendritic spines in pyramidal neurons at 2w (B), 3w (D) and 4w (F) post-birth 4. [file Image_2.TIF]

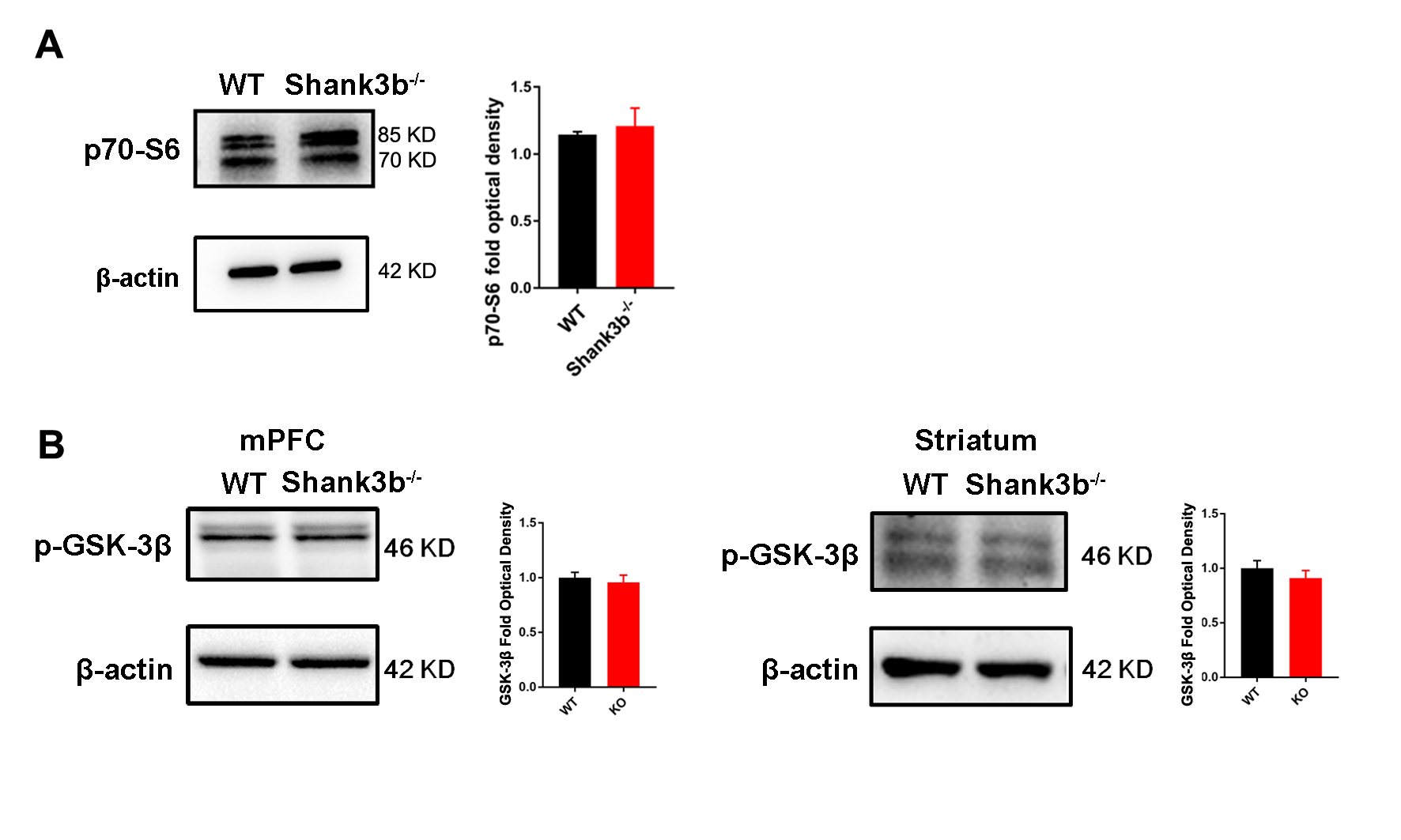

Supplement: FIGURE S3 — (A) Western blotting and quantification of p70-S6 in WT ACC and Shank3b−/− ACC. N = 6 mice per group. (B) Western-blotting and quantification of p-GSK-3βin the mPFC and striatum of WT and Shank3b−/− mice. N = 4 mice per group. [file Image_3.TIF]

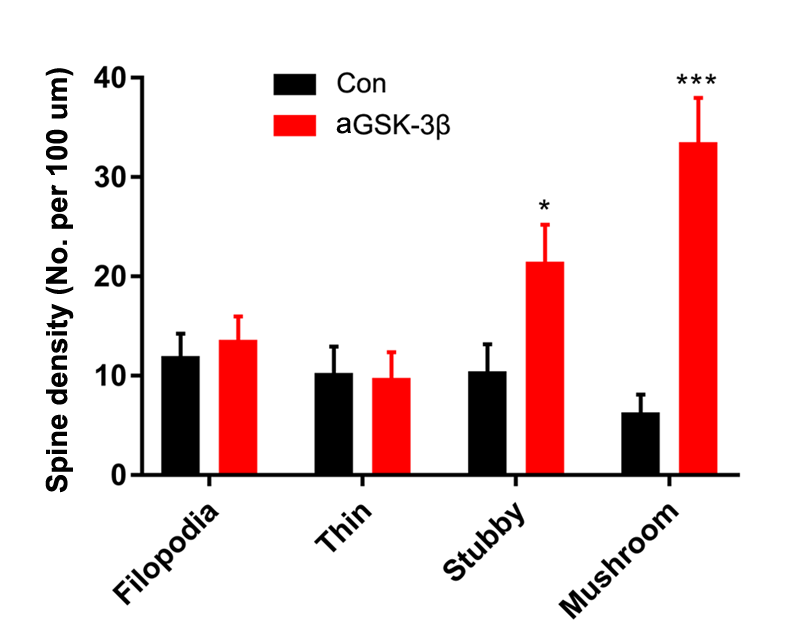

Supplement: FIGURE S4 — Densities of different types of basal spines of control and aGSK-3β treated Shank3b−/− ACC. Notice the increase of stubby and mushroom spines by aGSK-3β. Values represent mean ± SE. *P < 0.05, ***P < 0.001. [file Image_4.TIF]
